# Supplementary material for: AMPK modulation ameliorates dominant disease phenotypes of CTRP5 variant in retinal degeneration
Source: Commun Biol. 2021 Dec 9;4:1360. doi: 10.1038/s42003-021-02872-x (PMC8660775; doi:10.1038/s42003-021-02872-x)
Supplement: Supplementary file 2 — Supplementary Information [file 42003_2021_2872_MOESM2_ESM.pdf]

Supplementary Information for

AMPK modulation ameliorates dominant disease phenotypes of *CTRP5* variant in retinal degeneration

Table of Contents:

Supplementary Methods	pages 2-8
Supplementary Figures 1 to 10	pages 9-18
Supplementary Table 1	page 19
Supplementary References	pages 20-21

Supplementary Methods

iPSC Generation

Dissected pieces of skin biopsies were placed flat in 10 cm cell culture dishes underneath a coverslip with just enough Fibroblast media to cover the bottom of the dish and placed in 37 °C incubator. Fibroblast media consisted of 50% Feeder Media + 50% AminoMax II Complete Medium Supplement (ThermoFisher Scientific, Waltham, MA, #11-269-016). Media was added as necessary to compensate for evaporation until fibroblasts were confluent; at which point they were trypsinized with 0.25% trypsin/EDTA (ThermoFisher Scientific, #25200056) and passaged onto 6-well plates or T-25 flasks for expansion and cryopreservation.

Early passage (passage 0-2) fibroblasts were plated at low density and allowed to reach 70% confluency prior to reprogramming with Cytotune-iPS 2.0 Sendai Reprogramming kit (ThermoFisher Scientific, #A16517) according to manufacturer's instruction. After 3 weeks, cells were live-stained with TRA-1-60 mouse anti-human mAb, Alexa Fluor 555 conjugate (ThermoFisher Scientific, #A24874). Positive clones were marked with Nikon Object Marker and manually isolated and picked for clonal expansion on mouse embryonic feeders (MTI-GlobalStem, Gaithersburg, MD, #GSC-6001G). Feeder media consisted of Dulbecco's modified Eagle's medium (DMEM) high glucose no glutamine (ThermoFisher Scientific, #11960044) with 20% heat inactivated (30 min at 56 °C) fetal bovine serum (ThermoFisher Scientific, #10082147), 1X Penicillin-Streptomycin (ThermoFisher Scientific, #15140122), 2 mM L-Glutamine (ThermoFisher Scientific, #25030081), and 1mM Sodium-Pyruvate (ThermoFisher Scientific, #11360070).

iPSC media consisted of DMEM/F12 (ThermoFisher Scientific #11320-033) with 20% Knockout Serum Replacement (ThermoFisher Scientific #10828-028), 1X Non-Essential Amino Acids (ThermoFisher Scientific, #11140050), 1X Penicillin-Streptomycin (ThermoFisher Scientific, #15140122), 2 mM L-Glutamine (ThermoFisher Scientific, #25030081), and 55 µM 2-Mercaptoethanol (ThermoFisher Scientific, #21985023).

iPSC Media was supplemented daily with recombinant FGF (R&D Systems, Minneapolis, MN, #233-FB-025). When passaging ROCK inhibitor/Y-27632 (Tocris, Minneapolis, MN, #1254) was used to prevent disassociated cells from undergoing apoptosis. After several passages iPSCs were transferred to a feeder free protocol prior to differentiation [1].

Validation of iPSC Pluripotency

iPSCs were washed in DPBS (ThermoFisher Scientific, #14190-144) and fixed for 5 min with 4% paraformaldehyde (Electron Microscopy Sciences, Hatfield, PA, #15710) in PBST which consisted of 0.5% Tween-20 (Affymetrix, Santa Clara, CA, #900-64-5) in DPBS (ThermoFisher Scientific, #14190-144). Following fixation, cells were washed three times with PBST,

permeabilized with ICC buffer for 1 hour which consisted of DPBS, 0.5% Bovine Albumin (MP Biomedicals, Irvine, CA, #160069), 0.5% Tween-20, 0.1% Triton X-100 (Sigma, St. Louis, MO, #9002-64-5). Primary antibodies OCT4 (ThermoFisher Scientific, #MA1-104), NANOG (R&D Systems, #AF1997), SOX-2 (ThermoFisher Scientific, MA1-014), SSEA4 (Cell Signaling, Danvers, MA, #MC813) were added in ICC buffer and incubated overnight at 4 °C.

In vitro Embryoid Body Assay (stochastic differentiation into all 3 germ layers)

For the *in-vitro* embryoid body assay the following protocol was used to induce spontaneous differentiation. iPS cells were harvested by treating with 1 mg/mL collagenase type IV (ThermoFisher Scientific, #17104019) for 20 min at 37 ºC. Collagenase was aspirated and replaced with DMEM (ThermoFisher Scientific, #11960044). Colonies were scraped gently with a cell lifter (Corning, Corning, NY, #3008) and transferred to a 15 mL conical tube and allowed to settle to the bottom of the tube. Cell aggregates (detached colonies) were washed once in DMEM and allowed to settle again. Cell aggregates were then plated into ultra-low attachment 6-well plates for 24 h (Day 0) in 4-5 mL per well in iPSC media (w/ FGF-2) + 0.05% ROCK inhibitor/Y-27632 + 0.1% Matrigel (Corning, #356234) and left until Day 4 without media change. Day 5 media was changed by collecting all embryoid bodies (EBs) into a 15 mL conical tube and allowed to settle. Cells were resuspended in new media (no FGF-2) and supplemented with ROCK inhibitor/Y-27632. Day 7 EBs were trypsinized with 0.25% trypsin/EDTA (3-4 min) at 37 ºC. EBs were pipetted into single cells and approximately 5,000 cells were plated on Matrigel coated 24-well plates with 300 µL of media per well. On Day 8 an additional 200 µL of media was added to each well. Media was changed every other day from Day 9 to Day 18. On Day 18 cells were fixed with 4% PFA for 5 min and then stained for markers of the three germ layers: (1) endoderm: anti-AFP (ThermoFisher Scientific, #RB365A); (2) mesoderm: anti-Brachyury (R&D Systems, #AF2085) and anti-SMA (Sigma, #A5228); (3) ectoderm: anti-Nestin (EMD Millipore, Temecula, CA, #MAB5326) and TUJ1 (EMD Millipore, #MAB1637) with a standard IF protocol (see Immunostaining).

Karyotyping and short tandem repeat analysis

G-banded Karyotyping (to identify potential structural chromosomal abnormalities in pluripotent stem cell lines) and Short Tandem Repeat Analysis (STR Profiling 300 - to monitor changes in copy number variation and detect potential culture mix-ups/cross contamination of lines) were provided by Cell Line Genetics. The Ophthalmic Genomics Laboratory at the National Eye Institute, NIH performed Sanger Sequencing to confirm the disease-causing *C1QTNF5* variant (p.Ser163Arg) in L-ORD. The c.489C>G p.(Ser163Arg) variant in the *C1QTNF5* gene was assayed by Sanger sequencing using the BigDye Direct Cycle Sequencing Kit (ThermoFisher Scientific, #4458687) and a SeqStudio Genetic Analyzer (ThermoFisher Scientific, #A35644). The

M13-tagged PCR primers were GTAAAACGACGGCCAGTACTTTGCAGTGGGCACTAAGC and CAGGAAACAGCTATGACCGTCTGACGCACCCTTGCC.

Differentiation of iPSCs into RPE

iPSCs were differentiated into RPE using previously published protocols [1,2] with modifications. Briefly, iPSC colonies were seeded onto vitronectin (ThermoFisher Scientific, #A1700) coated 6-well plates in E8 media (ThermoFisher Scientific, #A1517001) supplemented with ROCK inhibitor/Y-27632 (Tocris Bioscience, #1254). After two days, cells reached confluency forming a monolayer and the E8 media was switched to differentiation media [DMEM/F12 (ThermoFisher Scientific, #11330032), N2 supplement (ThermoFisher Scientific, #A1370701), B27 (ThermoFisher Scientific, #17504044), KSR (ThermoFisher Scientific, #12618013), 20 ng/ml NOGGIN (R&D Systems, #6057), 5 μM CK1-7 Dihydrochloride (Sigma, #C0742), 5 μM SB 431542 hydrate (Sigma, #S4317), and 5 ng/ml IGF-1 (R&D Systems, #AFL291), 5 μM PD0325901 (Sigma, #PZ0612), 10 mM Nicotinamide (Sigma, #N0636), 150 ng/ml ACTIVIN A (R&D Systems, #338-AC/CF)]. Differentiating iPSCs that had committed to RPE cell fate were reseeded onto new vitronectin coated surfaces and maintained in RPE maintenance media (MEM+GlutaMAX, ThermoFisher Scientific, #32561037; 5% FBS, Hyclone, #SH30071.03; Taurine, Sigma, #T-0625; Thyronine, Sigma, #T-5516; Hydrocortisone, Sigma, #H-0396-10) for 15 days. RPE cells were enriched using negative selection with anti-CD24 (BD Biosciences, San Jose, CA, #655154) and anti-CD56 (BD Biosciences, #340723) antibodies and were seeded onto vitronectin coated transwells (Corning, #3460) and cultured for 6 weeks before any assays or experiments.

Cell Culture

iRPE were maintained in RPE maintenance media (5% serum) [3] which served as the culture medium for the experiments unless noted.

In silico analysis

The molecular modeling protein complex was built using a molecular visualization, modeling, and dynamics program, YASARA (www.yasara.com). The crystal structure for a human ADIPOR1 (chain A, residues 90-368) was extracted from the structure of the AdipoR1 adiponectin 1 receptor in complex with an Fv fragment (PDB ID:5lxg) [4,5]. A trimer of the C1q domains (residues 103-243) of the complement C1q tumor necrosis factor-related protein 5 (CTRP5) was used to build a trimeric heterocomplex with ADIPOR1 [6,7]. The structure of the trimeric ADIPOR1 was generated, optimized, and incorporated into a model of lipid membrane formed by 156x156 phosphatidyl-ethanolamine molecules. The structures of the CRTP5 Ser163 and Arg163 variants were independently docked to the subunits of the membrane-bound ADIPOR1 trimer. Atomic

structures of the wild type and mutant heterocomplexes were minimized iteratively to achieve a global energy minimum structure. The protein-membrane trimeric complexes were optimized and equilibrated using ~4 ns simulated annealing in water. A similar refinement was performed for p.Pro188Thr and p.Gly216Cys variants. The effect of the pathogenic variant on protein stability was evaluated as previously described [8,9].

Shapemetric Analysis Conversion Factors and No. of Cells Counted

Units in pixels are converted to real-world distance units with the following conversion:

For 20x, 1.291 pixels = 1 μm

For 40x, 2.595 pixels = 1 μm

For comparisons between healthy-iRPE and L-ORD iRPE: (cell area, **Figure 1**); (perimeter, major axis, minor axis, **Figure S5**), image analysis was performed on N= 14277 and N = 8284 cells respectively.

For comparisons between healthy-iRPE and L-ORD iRPE fed photoreceptor outer segments (with and without metformin treatment): (cell area, perimeter, major axis, minor axis, **Figure S6**), image analysis was performed on N= 688-1275 cells.

Staining of APOE Deposits Left on Transwells and Image Quantification

iRPE monolayers grown on transwells were incubated in sterile water (Sigma, #W3500-24X500ML) at room temperature for 5-10 minutes and carefully lysed by gentle pipetting. The transwells, now devoid of cells, were fixed in 4% PFA for 10 min, washed 3x in PBST (1xPBS, 0.5% Tween20) and permeabilized in ICC blocking buffer (1x PBS, 1% BSA, 0.25% Tween20, 0.25% TritonX-100) for 1 h. Transwells were incubated overnight at room temperature with an antibody for APOE (1:100, EMD Millipore, #AB947) diluted in ICC blocking buffer. Cells were washed 3x in ICC buffer and Hoechst 33342 was added at 1:2000 dilution. Samples were washed 3x in ICC blocking buffer and mounted onto glass slides using Fluoromount-G aqueous mounting medium (Southern Biotech, Birmingham, AL, #0100-01). Images were acquired on a Nikon A1R upright fluorescent microscope with using Nikon Elements software (Nikon). Image quantification was performed in ImageJ on original unprocessed images. 25x images of APOE-stained transwells were split into their individual color channels. The red (APOE) and blue (Hoechst) channels were exported as to 16-bit monochromatic images. To remove unlysed cellular material from the APOE quantification, a mask was created from the blue channel and multiplied to the background corrected red channel using Image Calculator (ImageJ). The total brightness of the remaining red channel given in artificial densitometric units (ADU) was measured from the resultant image.

Statistical analysis of APOE total brightness was performed using student's t-test.

Cryosectioning (APOE-stained iRPE)

Healthy and L-ORD-iRPE were fed POS for 7 consecutive days in either RPE media (5% serum) or in RPE media containing either 1 μM ara-A or 3 mM metformin.

Immunofluorescent samples stained for APOE, Collagen IV, and counterstained with Hoechst 33342 were washed 3 times in 1x PBS and placed in 10% sucrose. After one hour the samples were removed and placed in 20% sucrose for another hour. Lastly, the samples were placed into 30% sucrose overnight. Samples were taken to a cryosection device (Leica) and embedded in Tissue-Tek optimal cutting temperature (O.C.T) compound (Sakura Finetek, Torrance, CA). Sections were cut to 12 μm thickness and transferred to and mounted onto glass slides using Fluoromount-G aqueous mounting medium (Southern Biotech, #0100-01). Images were taken on a Zeiss Axio Imager M2 inverted fluorescent microscope with Apotome 2 and Zen 2012 software (Carl Zeiss AG) at 20x, 63x, and 100x. Images were also taken on a Nikon A1R at 60x with 6x digital zoom. Embedded tissues were stored at -80 ºC.

Image quantification of APOE Cryosections

Image quantification was performed in ImageJ. 20x images of successfully cut iRPE on transwells were split into their individual color channels. The red channel corresponding to APOE staining was converted to 16-bit grayscale. Freehand selections of the ROI were drawn outlining the apical APOE profile and duplicated using ROI manager to outline the basal APOE profile. The integrated density (the product of area and mean gray value) was measured for each defined ROI.

Lipid Extraction Prior to Mass Spectrometry

Samples were collected daily following 4 hours or 24 hours of feeding ~10 photoreceptors outer segments (InVision BioResources, #98740) / RPE cell or following 24 h of 32:6 (Cayman Chemical, #10497, 1 μM) and 34:6 (Cayman Chemical, #10539, 1 μM) in 0.5% serum containing media. Then the media was collected daily on ice from 12 transwells for 5-6 consecutive days. The conditioned media was centrifuged at 3000 g/15 min and supernatants transferred to 15 mL tubes, frozen on dry ice and stored at -80 ºC.

The medium samples were lipid extracted using a modified Bligh-Dyer method as follows: after thawing the medium samples, they were added 2.5 x volumes of cold MeOH and placed on ice for 30 min and then centrifuged. Then the supernatants were added with 1.25 volumes of $CHCl_3$ and an internal standard mixture: (Cayman Chemical, LTB4-d4, #29629; PGD2-d4, #10007272; 15-HETE-d8, #334720; AA-d8, #390010; and EPA-d5, #27358; Cayman Chemical) was added. After sonication in the water bath with ice for 30 min, the samples were stored at -80 °C

overnight. Then samples were added 1.25 x volumes of $CHCl_3$, and 1.25 x volumes of pH 3.5 H_2O. After the phase separation, the upper phase was removed, and the bottom phase was dried under a gentle stream of nitrogen. The samples were re-suspended in 30 μL of MeOH:H_2O 1:1 for mass spectrometry.

Fluorogenic method for determination of Ceramidase Activity

Ceramidase activity was measured as described in Beida et. al.[12] Briefly iRPE cultured on transwells were washed twice with PBS and incubated in trypsin-EDTA (0.25%) (ThermoFisher Scientific Cat#: 25200056) at 37°C for 45 minutes. Cells were collected in 5% serum containing RPE media and centrifuged at 12000g for 5 minutes. Cell pellets were resuspended in 100μl of a 0.2M sucrose solution and sonicated (Q125, QSonica) on ice using three cycles of 5s sonication (50% amplitude) with 5s resting on ice. Total sonication time was 15s. Cell homogenates were than centrifuged for 15,000g for 3min. The supernatant was collected and used for protein quantification. The enzymatic assay was performed in 96 well plates (Corning, Cat#: 29442-314). Each well contained a mixture of 74.5 μl of 25mM sodium acetate buffer pH 4.5 (Sigma, Cat#: S2889-250G), 0.5 μl of 4mM Rbm14-12 (Avanti Polar Lipids, Cat#: RBM14C12) substrate solution in ethanol (Sigma, E7023-500ML), and 25uL of the protein samples obtained from each transwell sample (each sample was first diluted as follows: 60 μl sample + 15 μl 0.2M sucrose). The negative control consisted of absence of protein extracts. The plate was incubated at 37°C with no agitation, without CO2. The enzymatic reaction was stopped by adding 50 μl of methanol (Sigma, Cat#: 322415-100ML), and 100 μl of a 2.5 mg/ml NaIO4 solution (Sigma, Cat#: 311448-5G) in 100mM glycine (Sigma, Cat#: 50046-50G) /NaOH (Sigma, Cat#: 2105-50ML) buffered to a pH of 10.6 was added to each well to release the umbelliferone from the Rbm14-12 substrate and to maximize the umbelliferone's fluorescence. The plate was protected from light for 1 hour and the released fluorescence was quantified using a microplate reader (ex 360 nm, em 446 nm). The standard curve of umbelliferone (Sigma, Cat#: H24003-25G) fluorescence was made as follows:

The umbeilliferone stock was diluted in ethanol at 5mM and the first concentration of the calibration curve was 10μm in sodium acetate buffer (100μl). 1/2 serial dilutions were made using sodium acetate buffer until 8 points on the standard curve were obtained. BCA (Pierce BCA protein assay kit, ThermoScientific, Cat#: 23225) was performed to quantify protein content in each transwell sample per manufacturer's instructions. Samples were diluted 5-fold prior to running the BCA assay. After normalizing the data per well based on protein content the final enzyme activity is given in pmol/h/mg.

Western Blot Analysis of CTRP5

Western blots of CTRP5 performed on the insoluble pellets remaining after centrifugation of apical conditioned media. The media from the apical side of the transwells were collected on ice 72 h after the last media change. The conditioned media was centrifuged at 13,200 rpm at 4 ºC for 15 min. The supernatant was transferred to fresh 1.5 mL Eppendorf tubes and frozen on dry ice and stored at -80 ºC. The amount of soluble protein secreted (CTRP5) by the cells was normalized to the total protein of the cell lysate (indicated by β-actin).

Supplementary Figure 1. Related to Figure 1. G-banded Karyotype analysis of iPSC lines derived from healthy donors and L-ORD patients. All iPSC lines displayed a normal karyotype (46, XY or XX).

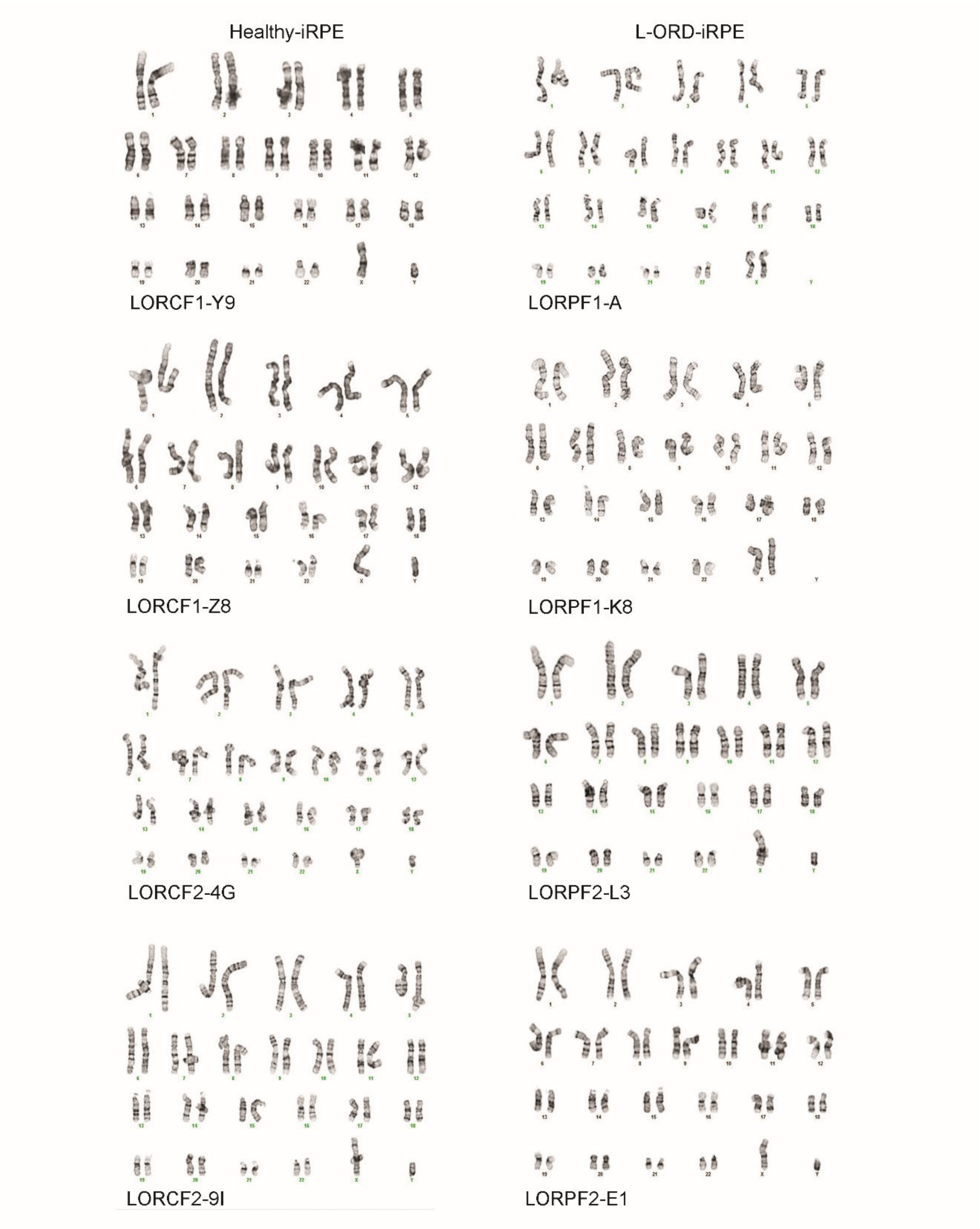

Supplementary Figure 2. Related to Figure 1. (a) Confocal images of MFRP and EZRIN expression in healthy and L-ORD-iRPE indicate no patient-specific differences in expression. Hoechst nuclei marker (blue), MFRP (green), EZRIN (apical marker, magenta). Scalebar: 10 µm. (b) Low magnification SEM images of healthy and L-ORD-iRPE; both show normal hexagonal appearance, but L-ORD-iRPE appear to be larger in size. Scalebar: 2 µm. (c) Quantification of images of APOE-stained transwells after cells removed by lysis with water expressed in artificial densitometric units (A.D.U.). L-ORD-iRPE (*n*=14); healthy-iRPE (*n*=11). ** $p<0.01$

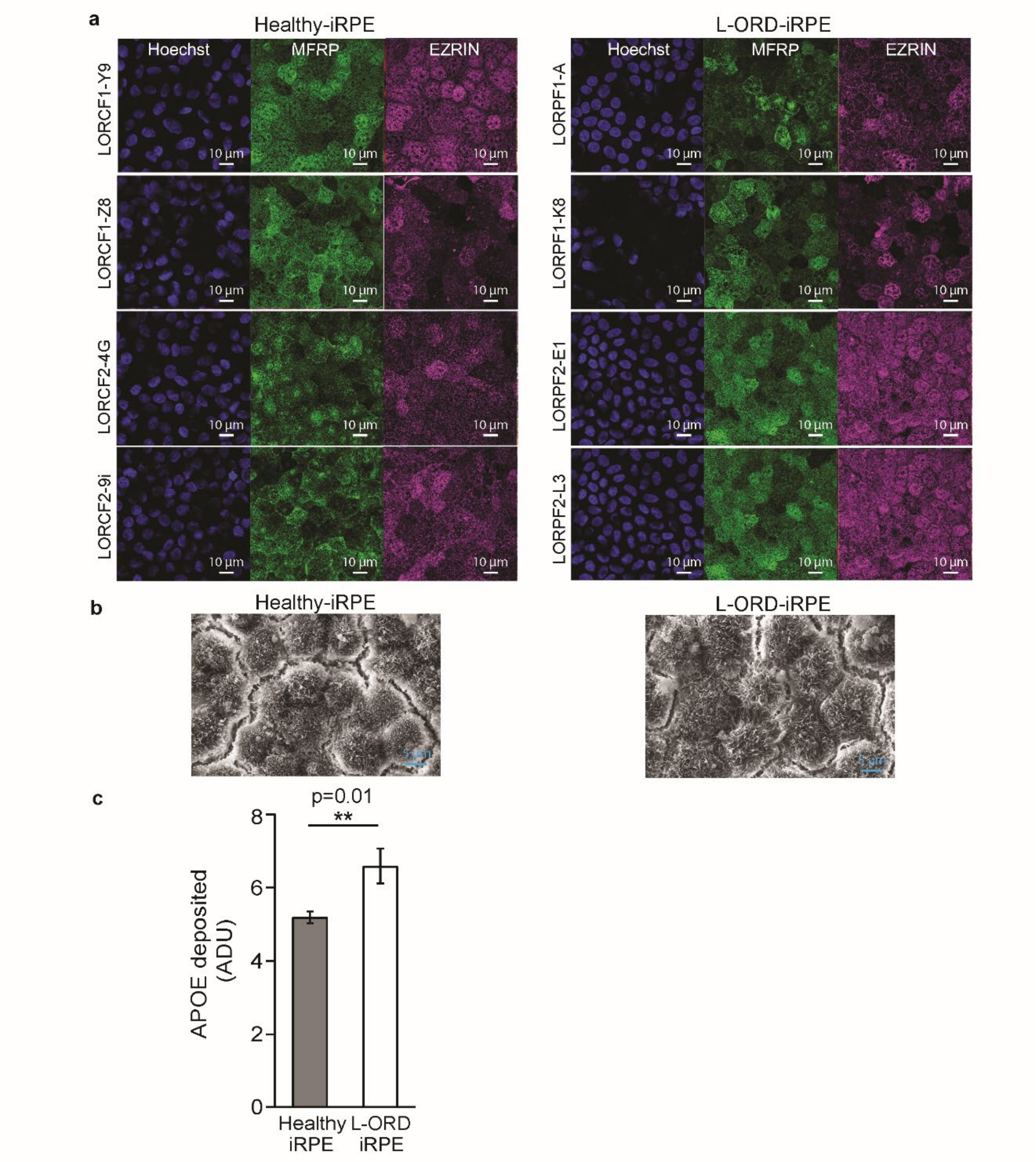

Supplementary Figure 3. Related to Figure 2. (a) Boxplots of ΔCt values for *CTRP5* and *MFRP* transcripts in healthy-iRPE and L-ORD-iRPE are comparable. *CTRP5* (healthy-iRPE: *n*=10; L-ORD-iRPE: *n*=6), *MFRP* (healthy-iRPE: *n*= 9; L-ORD-iRPE: *n*=6). Normalized to reference gene: *RPL13A*. The box top and bottom define the 75th and 25th percentiles and the whisker top and bottom define the 90th and 10th percentiles. The horizontal line within the boxplot defines the median. (b) Overexpression of V5-tagged WT-CTRP5 (green) alone doesn't lead to cytoplasmic retention. (c) Immunofluorescent images of iRPE stained for CTRP5 (red), CALRETICULIN (green) in L-ORD and Healthy-iRPE, and Hoechst (blue) Scale bar: 10 µm. (d) Heatmap of expression (ΔCt) of ER-stress reporters (*HERPUD1*, *PDIA4*, *EDEM1*, *XBP1*, *SQSTM1*) in L-ORD and healthy-iRPE are comparable. Normalized to housekeeping gene: *HPRT1*. (e) Immunofluorescent images of healthy and L-ORD-iRPE monolayers costained with CTRP5 (green) and EEA1 (red) in healthy and L-ORD-iRPE Scale bar: 10 µm. (f) Confocal images of L-ORD-iRPE treated with and without Bafilomycin A1 for 3 hours and then fixed and stained for CTRP5 (red) and LAMP1 (green). Pearson's colocalization coefficient – healthy-iRPE CTRP5/LAMP1 (untreated: 0.27± 0.06; BafA1 treated: 0.35±0.12), L-ORD-iRPE (untreated: 0.26± 0.14; BafA1 treated: 0.32±0.08). (g) Cross-section view of Immunofluorescent images of L-ORD-iRPE monolayers stained for CTRP5 (red). F-ACTON (white), nucleus (blue). Scale bar: 10 µm.

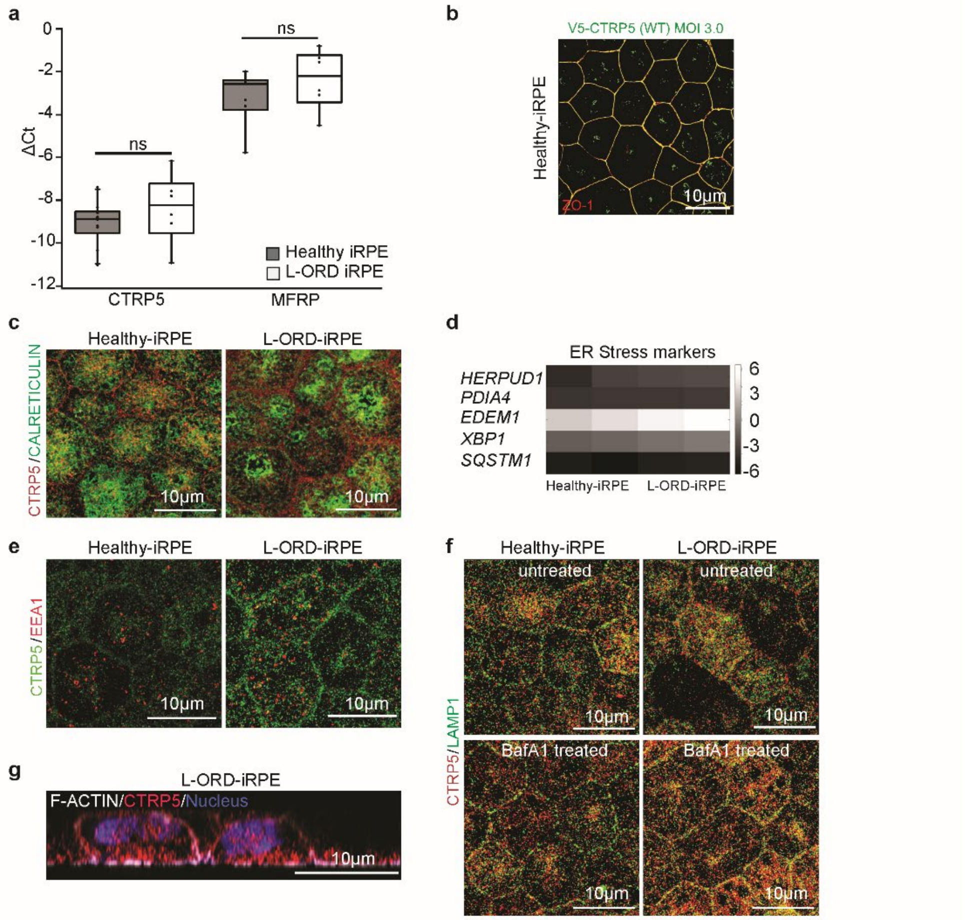

Supplementary Figure 4. Related to Figure 2. (a, b) Immunofluorescent staining for ADIPOR1 (a, green) and ADIPOR2 (b, red) are comparable in healthy and L-ORD iRPE. Representative X-Z cross sections of the 3-D image stack are shown for each donor. At least two images were taken per donor. Nuclei stained with Hoechst 33342. Scale bar: 5 µm. (c) Box plot showing 1.9-fold higher ADIPOR1 gene expression in healthy-iRPE compared to L-ORD-iRPE. ΔCt normalized to *HPRT1*. Healthy-iRPE (*n*=6), L-ORD-iRPE (*n*=6). (d) Western blot of ADIPOR1 (*) expression in L-ORD and healthy-iRPE. GAPDH was used as the loading control. (e) Quantification of the Western blot shown in (D), showing a 1.8-fold increase in ADIPOR1 protein expression in healthy-iRPE compared to L-ORD-iRPE. Healthy-iRPE (*n*=4), L-ORD-iRPE (*n*=4). (f, g) *In silico* model of CTRP5 disease-associated substitutions illustrate their close position to the docking interface with ADIPOR1 and predict a change in hydrophobic and repulsive interactions with residues on ADIPOR1. *p<0.05, ns = non-significant, A.U. = arbitrary units

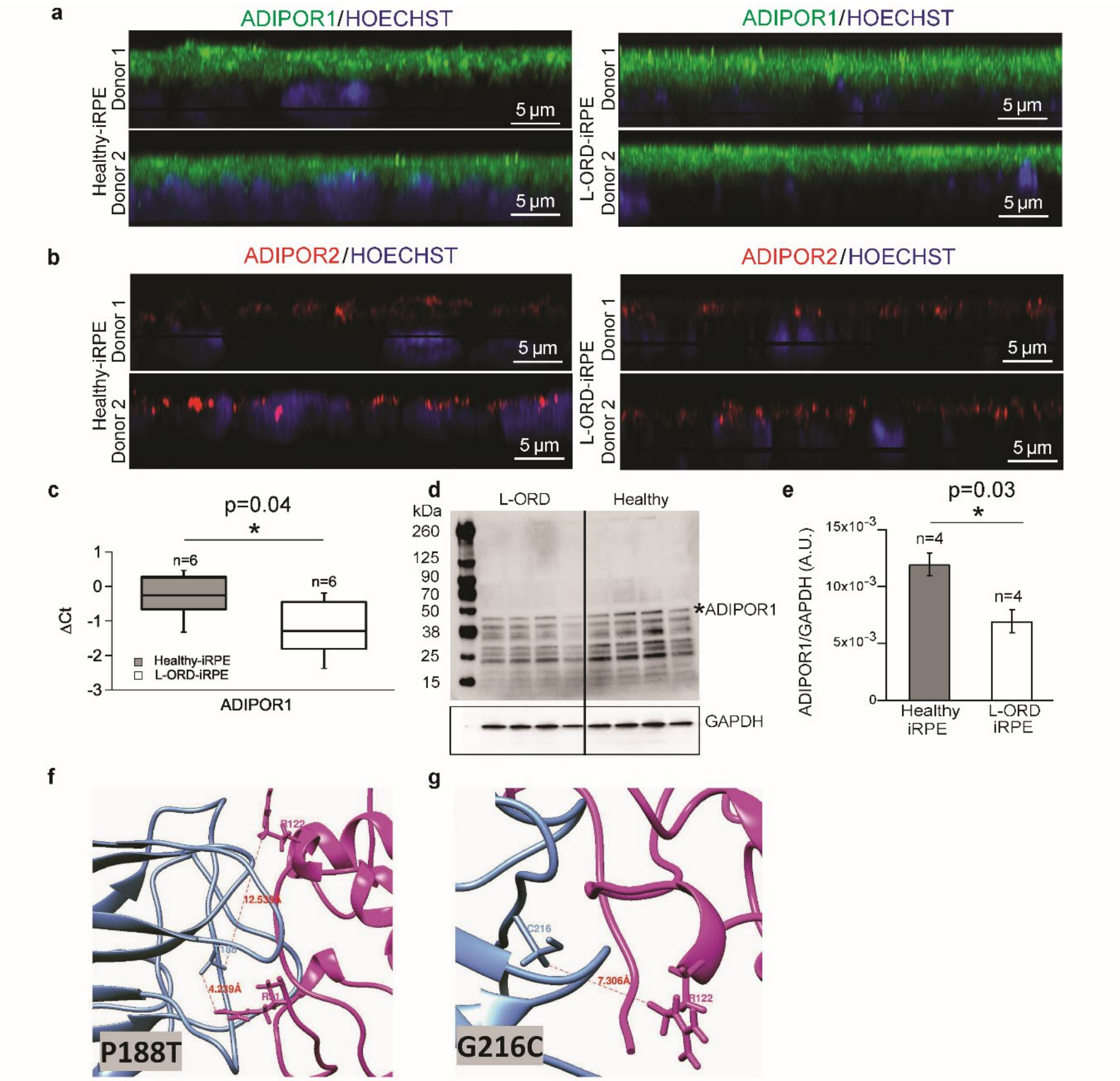

Supplementary Figure 5. Related to Figure 4. (a) Ceramide (red) is expressed in both healthy-iRPE and L-ORD-iRPE and localizes to the base of the primary cilia labeled with ARL13B (green). The primary cilia, is an important sensory organelle that is essential for RPE maturation[10] and has been shown to be regulated by ceramide[11]. Note there was no difference in the amount of ceramide synthesized in healthy and L-ORD-iRPE. Scale bar: 10 µm. (b) Box plot of measured ceramidase activity of healthy-iRPE (*n*=6) and L-ORD-iRPE (*n*=6) shows no difference. (c) Lipidomic analysis of apical media collected from healthy and L-ORD-iRPE under normal cell culture conditions and after being subjected to daily POS feeding. POS feeding increases apical secretion of DHA compared to AA or EPA. Healthy-iRPE secrete ~25% more DHA than L-ORD-iRPE. ns = non-significant

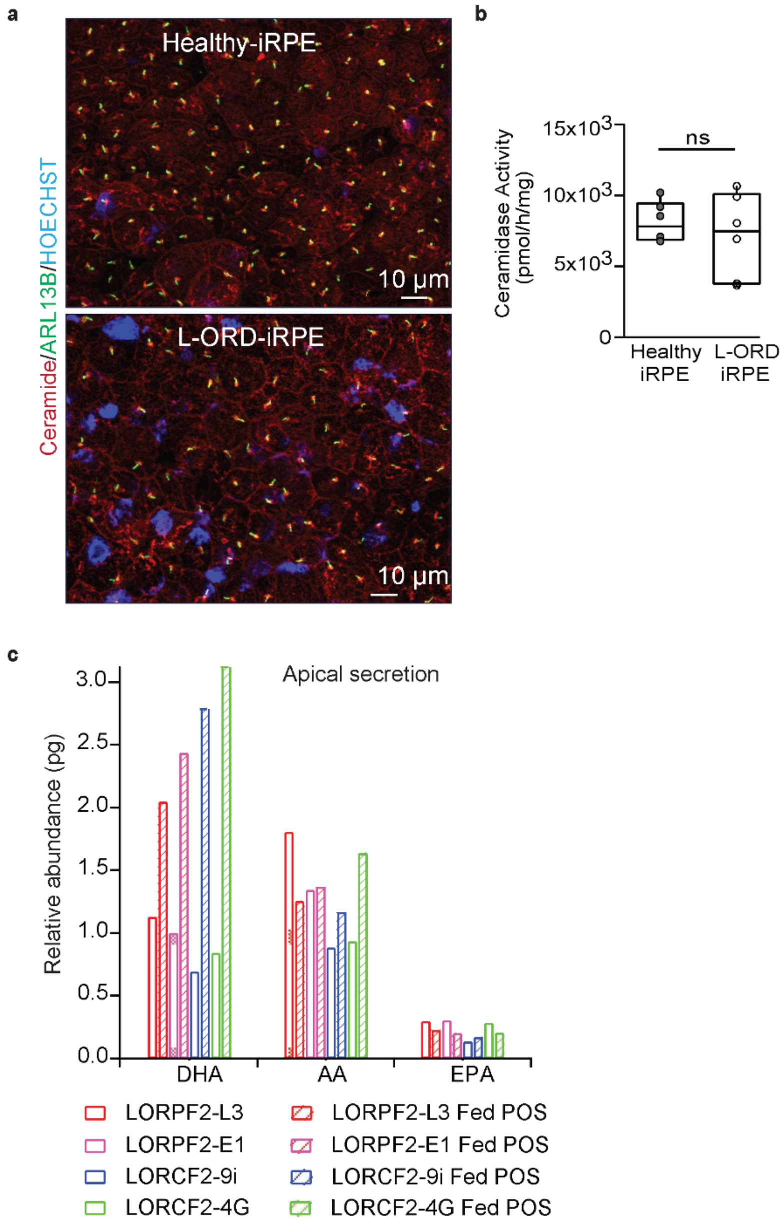

Supplementary Figure 6. Related to Figure 1 to 4. (a, b) Green fluorescent protein (GFP) was used to track the transduction efficiency of lentivirus expressing a WT-CTRP5 construct. Three different multiplicities of infection (MOI) 0.5, 1.5, 3.5 were used. GFP expression monitored of days 4 and 14 post-transduction. (c, d) Apical and basal CTRP5 secretion as measured by ELISA in L-ORD-iRPE 14 days after transduction with increasing MOI (0.5, 1.5, 3.0) of WT-CTRP5 expressing lentiviral constructs. (*n*=4 per MOI). (e) pAMPK levels determined by ELISA in L-ORD-iRPE with increasing MOI (0.5, 1.5, 3.0) of lentiviral constructs expressing WT-CTRP5 (*n*=3 per MOI). (f) Apical and basal VEGF secretion in L-ORD-iRPE with increasing MOI (0.5, 1.5, 3.0) of lentivirus expression WT-CTRP5, as measured by ELISA (*n*=4 each MOI). * p<0.05, ** p<0.01, *** p<0.0001, A.U. = arbitrary units.

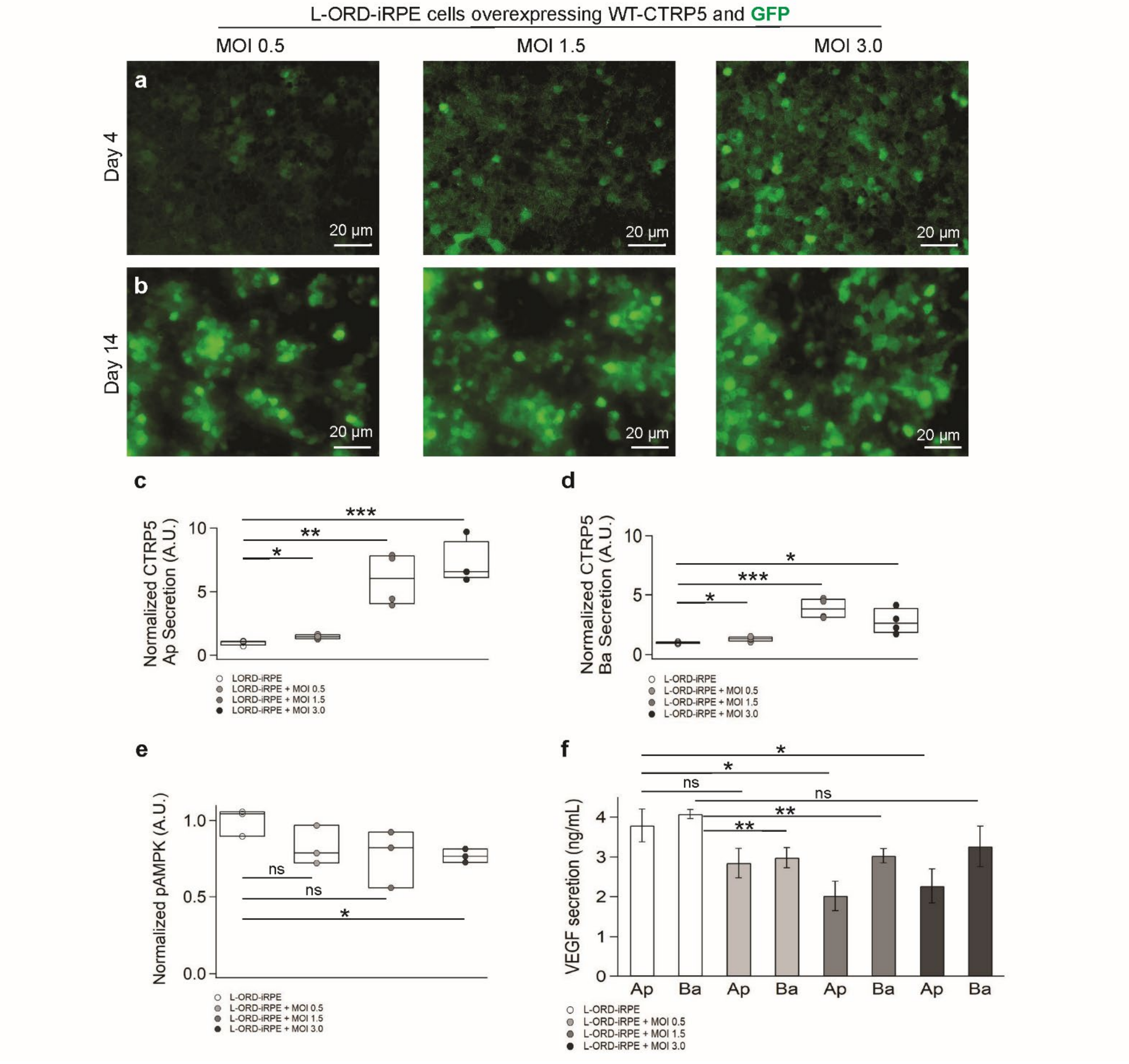

Figure S7. Related to Figure 5. Shapemetric analysis of healthy and L-ORD-iRPE under basal conditions: (A) Perimeter (B) Major axis (C) Minor axis. ***p<0.001.

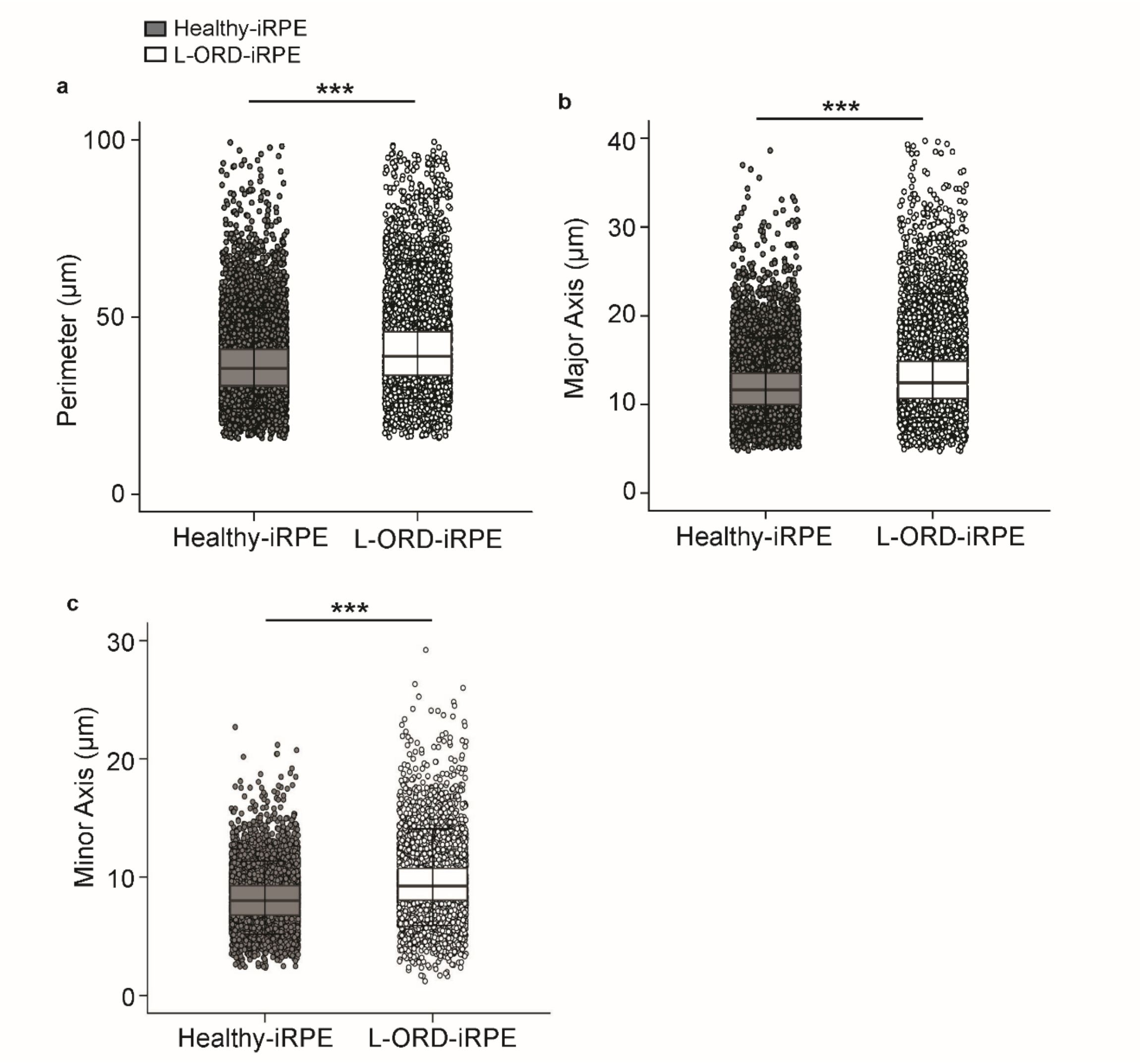

Figure S8. Related to Figure 5. Shapemetric analysis of healthy and L-ORD-iRPE following 7-day POS feeding and metformin treatment. Metformin treatment mitigates the POS-induced cell morphometry changes in L-ORD-iRPE: (A) Area (B) Perimeter (C) Major axis (D) Minor axis. ***p<0.001, ns = non-significant

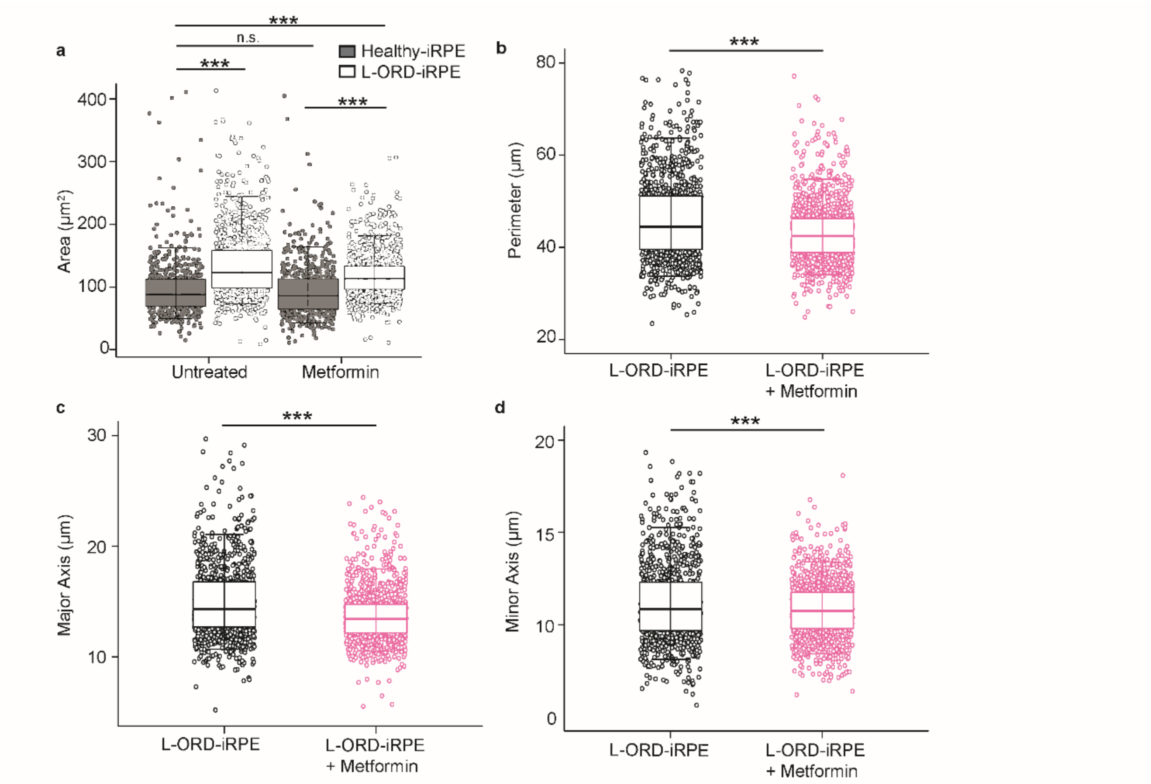

Supplementary Figure 9 Related to Figure 5. (a) Box plot showing several key metabolic genes (*HMGCS, PEDF-R*, *PRKAG1*) altered by metformin treatment in L-ORD-iRPE. ΔCt normalized to *HPRT1* and *B2M*. L-ORD-iRPE (*n*=5), L-ORD-iRPE+metformin (*n*=4). *p<0.05, **p<0.01.

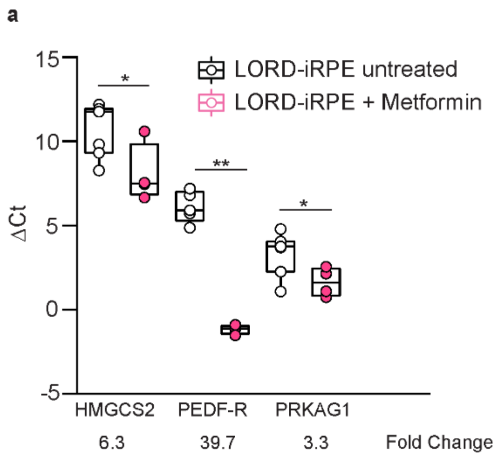

Supplementary Figure 10 Uncropped Western Blots.

Figure 3e

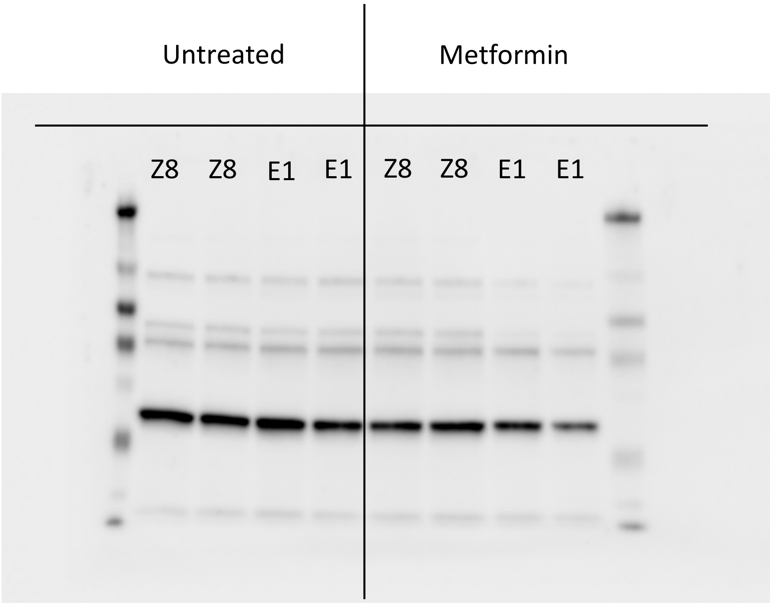

Figure 3g

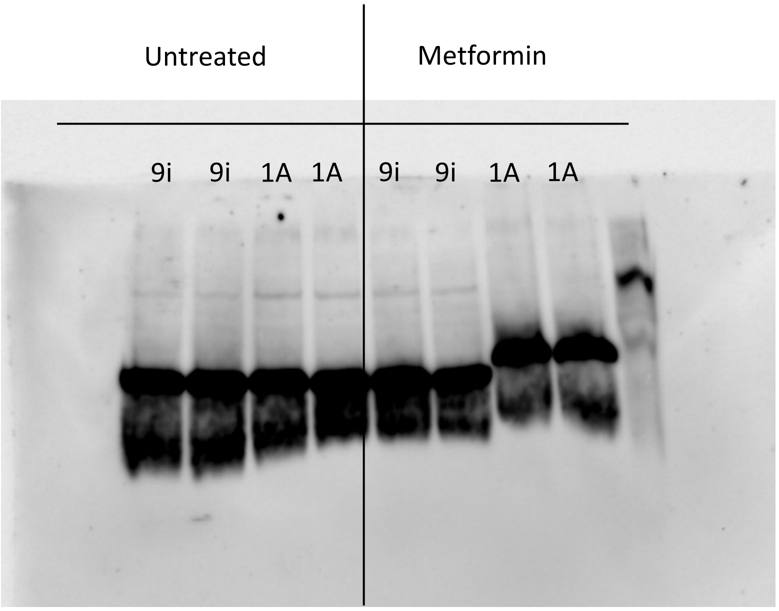

Figure 6d

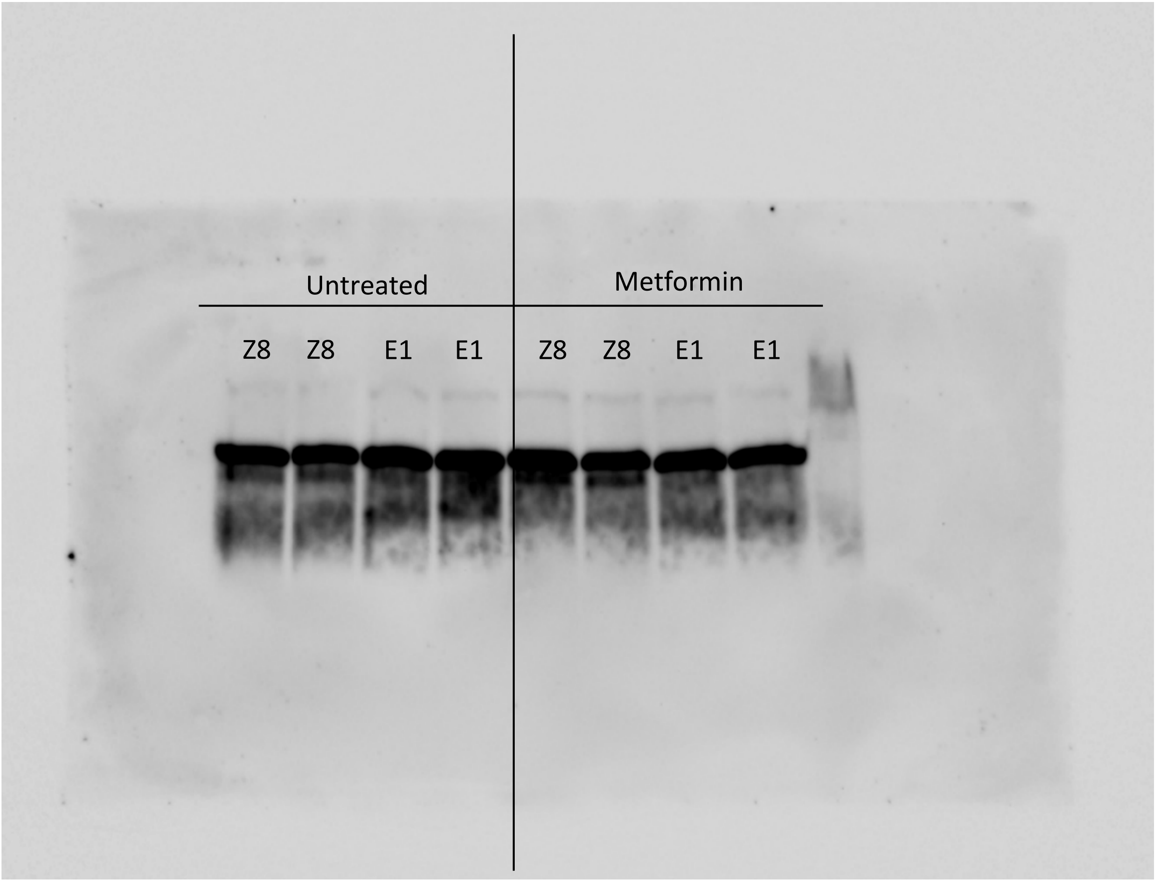

Supplementary Table 1. List of eight four dedifferentiation genes used for analysis of L-ORD-iRPE cells

AHNAK	*COL1A2*	*FGFBP1*	*ILK*	*MMP3*	*PPPDE2*	*SNAI3*	*TGFB3*	*WNT5A*
AKT1	*COL3A1*	*FN1*	*ITAGA5*	*MMP9*	*PTK2*	*SOX10*	*TIMP1*	*WNT5B*
BMP1	*COL5A2*	*FOXC2*	*ITGAV*	*MSN*	*PTP4A1*	*SPARC*	*TMEFF1*	*ZEB1*
BMP2	*CTNNB1*	*FZD7*	*ITGB1*	*MST1R*	*RAC1*	*SPP1*	*TMEM132A*	*ZEB2*
BMP7	*DSC2*	*GNG11*	*JAG1*	*NODAL*	*RGS2*	*STAT3*	*TSPAN13*	
CALD1	*DSP*	*GSC*	*KRT14*	*NOTCH1*	*RPLP0*	*STEAP1*	*TWIST1*	
CAMK2N	*EGFR*	*GSK3B*	*KRT19*	*NUDT13*	*SERPINE*	*TCF4*	*VCAN*	
CAV2	*ERBB3*	*GUSB*	*KRT7*	*OCLN*	*SMAD2*	*TFPI2*	*VIM*	
CDH1	*ESR1*	*IGFBP4*	*MAP1B*	*PDGFRB*	*SNAI1*	*TGFB1*	*VPS13A*	
CDH2	*F11R*	*IL1RN*	*MMP2*	*PLEK2*	*SNAI2*	*TGFB2*	*WNT11*	

Supplementary References

1 Sharma, R. *et al.* Clinical-grade stem cell-derived retinal pigment epithelium patch rescues retinal degeneration in rodents and pigs. *Science Translational Medicine* **11**, doi:10.1126/scitranslmed.aat5580 (2019).

2 Ferrer, M. *et al.* A multiplex high-throughput gene expression assay to simultaneously detect disease and functional markers in induced pluripotent stem cell-derived retinal pigment epithelium. *Stem Cells Transl Med* **3**, 911-922, doi:10.5966/sctm.2013-0192 (2014).

3 Maminishkis, A. *et al.* Confluent monolayers of cultured human fetal retinal pigment epithelium exhibit morphology and physiology of native tissue. *Invest Ophthalmol Vis Sci* **47**, 3612-3624, doi:10.1167/iovs.05-1622 (2006).

4 Tanabe, H. *et al.* Crystal structures of the human adiponectin receptors. *Nature* **520**, 312-316, doi:10.1038/nature14301 (2015).

5 Vasiliauskaité-Brooks, I. *et al.* Structural insights into adiponectin receptors suggest ceramidase activity. *Nature* **544**, 120-123, doi:10.1038/nature21714 (2017).

6 Tu, X. & Palczewski, K. Crystal structure of the globular domain of C1QTNF5: Implications for late-onset retinal macular degeneration. *J Struct Biol* **180**, 439-446, doi:10.1016/j.jsb.2012.07.011 (2012).

7 Tu, X. & Palczewski, K. The macular degeneration-linked C1QTNF5 (S163) mutation causes higher-order structural rearrangements. *J Struct Biol* **186**, 86-94, doi:10.1016/j.jsb.2014.02.001 (2014).

8 McCafferty, C. L. & Sergeev, Y. V. In silico Mapping of Protein Unfolding Mutations for Inherited Disease. *Sci Rep* **6**, 37298, doi:10.1038/srep37298 (2016).

9 Ortiz, F. W. & Sergeev, Y. V. Global computational mutagenesis of domain structures associated with inherited eye disease. *Sci Rep* **9**, 3676, doi:10.1038/s41598-019-39905-9 (2019).

10 May-Simera, H. L. *et al.* Primary Cilium-Mediated Retinal Pigment Epithelium Maturation Is Disrupted in Ciliopathy Patient Cells. *Cell Rep* **22**, 189-205, doi:10.1016/j.celrep.2017.12.038 (2018).

11 Wang, G., Krishnamurthy, K. & Bieberich, E. Regulation of primary cilia formation by ceramide. *J Lipid Res* **50**, 2103-2110, doi:10.1194/jlr.M900097-JLR200 (2009).

12. Bedia, C., L. Camacho, J. L. Abad, G. Fabriàs & T. Levade (2010) A simple fluorogenic method for determination of acid ceramidase activity and diagnosis of Farber disease. *J Lipid Res,* 51**,** 3542-7.
