# Supplementary material for: AMPK modulation ameliorates dominant disease phenotypes of CTRP5 variant in retinal degeneration
Source: Commun Biol. 2021 Dec 9;4:1360. doi: 10.1038/s42003-021-02872-x (PMC8660775; doi:10.1038/s42003-021-02872-x)
Supplement: Supplementary file 3 — Description of Additional Supplementary Files [file 42003_2021_2872_MOESM3_ESM.pdf]

Description of Additional Supplementary Files

File name: Supplementary Data

Description: All source data underlying graphs and charts is supplied in Supplementary Data -

Fig 1f. VEGF ELISA (Ap, Ba secretion)

Fig 2a,b. CTRP5 ELISA (Ap, Ba secretion)

Fig 3a. pAMPK levels (baseline)

Fig 3b. pAMPK levels in iPSC-RPE (5% and 0% serum)

Fig 3d. pAMPK levels in iPSC-RPE (0% serum + AICAR or BAM15)

Fig 3e-f. WB analysis of PGC1α

Fig 3g-h. WB analysis of phospho-PGC1α

Fig 3j. VEGF ELISA (iPSC-RPE treated with ara-A)

Fig 4c. PEDF ELISA (baseline)

Fig 4d. Phospholipase A2 activity in iPSC-RPE (baseline)

Fig 4e. Phospholipase A2 activity in iPSC-RPE under conditions of elevated pAMPK (0% serum)

Fig 4f-g. Seahorse XF Cell Mito Stress Test Analysis

Fig 4h. Summary of Lipidomic Analysis of NPD1 apical secretion

Fig 4i. Phagocytosis measurement by flow cytometry

Fig 5g. Measure of apical secretion of β-hB

Fig 5h. Lipidomic analysis of NPD1 apical secretion (iPSC-RPE treated with Metformin)

Fig 6d. WB analysis of APOE (cell lysate)

Fig 6e. Image analysis of subcellular APOE deposition

Fig 6f. VEGF ELISA (Ap, Ba secretion) treated with Metformin

Fig 6g. pAMPK levels in iPSC-RPE (0% serum + AICAR) treated with Metformin

Supplementary Figure 2c. Image analysis: APOE deposited on transwells

Supplementary Figure 3a. Gene expression of *CTRP5* and *MFRP*

Supplementary Figure 4d. ADIPOR1 WB analysis

Supplementary Figure 5a. *ADIPOR1* gene expression analysis

Supplementary Figure 5b. Ceramidase Assay

Supplementary Figure 6c-d. CTRP5 Elisa – CTRP5 overexpression study

Supplementary Figure 6e. pAMPK Elisa – CTRP5 overexpression study

Supplementary Figure 6f. VEGF Elisa – CTRP5 overexpression study
