# Supplementary material for: AMPK modulation ameliorates dominant disease phenotypes of CTRP5 variant in retinal degeneration
Source: Commun Biol. 2021 Dec 9;4:1360. doi: 10.1038/s42003-021-02872-x (PMC8660775; doi:10.1038/s42003-021-02872-x)
Supplement: Supplementary file 5 — Reporting Summary [file 42003_2021_2872_MOESM5_ESM.pdf]

nature research

Corresponding author(s): Dr. Kapil Bharti
COMMSBIO-20-2623A

Last updated by author(s): Oct 30, 2021

Reporting Summary

Nature Research wishes to improve the reproducibility of the work that we publish. This form provides structure for consistency and transparency in reporting. For further information on Nature Research policies, see our Editorial Policies and the Editorial Policy Checklist.

Statistics

For all statistical analyses, confirm that the following items are present in the figure legend, table legend, main text, or Methods section.

n/a	Confirmed	
☐	☒	The exact sample size (n) for each experimental group/condition, given as a discrete number and unit of measurement
☐	☒	A statement on whether measurements were taken from distinct samples or whether the same sample was measured repeatedly
☐	☒	The statistical test(s) used AND whether they are one- or two-sided<br>*Only common tests should be described solely by name; describe more complex techniques in the Methods section.*
☒	☐	A description of all covariates tested
☒	☐	A description of any assumptions or corrections, such as tests of normality and adjustment for multiple comparisons
☐	☒	A full description of the statistical parameters including central tendency (e.g. means) or other basic estimates (e.g. regression coefficient) AND variation (e.g. standard deviation) or associated estimates of uncertainty (e.g. confidence intervals)
☒	☐	For null hypothesis testing, the test statistic (e.g. F, t, r) with confidence intervals, effect sizes, degrees of freedom and P value noted<br>*Give P values as exact values whenever suitable.*
☒	☐	For Bayesian analysis, information on the choice of priors and Markov chain Monte Carlo settings
☒	☐	For hierarchical and complex designs, identification of the appropriate level for tests and full reporting of outcomes
☐	☒	Estimates of effect sizes (e.g. Cohen's d, Pearson's r), indicating how they were calculated

Our web collection on statistics for biologists contains articles on many of the points above.

Software and code

Policy information about availability of computer code

Data collection	Commercial software: Zen 3.2 (blue edition) (Zeiss), NIS Elements AR (Nikon),
Data analysis	MatLab R2021a, ImageJ 1.53c, Agilent Seahorse Wave Desktop, Microsoft Excel, Bio-rad Image Lab 6.1

For manuscripts utilizing custom algorithms or software that are central to the research but not yet described in published literature, software must be made available to editors and reviewers. We strongly encourage code deposition in a community repository (e.g. GitHub). See the Nature Research guidelines for submitting code & software for further information.

Data

Policy information about availability of data

All manuscripts must include a data availability statement. This statement should provide the following information, where applicable:

- Accession codes, unique identifiers, or web links for publicly available datasets
- A list of figures that have associated raw data
- A description of any restrictions on data availability

All the processed data are available within the article. Most of the raw data generated in this study are provided in the Supplementary Information/Source Data file. The data that support the findings of this study are available from the corresponding author upon reasonable request.

Field-specific reporting

Life sciences study design

All studies must disclose on these points even when the disclosure is negative.

Sample size	Sample size was determined upon availability, and chosen based on previous experience and previous studies. No statistical test was performed prior the experiment to select the sample size.
Data exclusions	No data were excluded
Replication	All experiments were performed a minimum of three times or were pooled from multiple independent samples and yielded similar results.
Randomization	No randomization was required because all the samples were uniformly acquired
Blinding	Image analysis and quantification was performed using automated scripts to prevent biased quantification

Reporting for specific materials, systems and methods

We require information from authors about some types of materials, experimental systems and methods used in many studies. Here, indicate whether each material, system or method listed is relevant to your study. If you are not sure if a list item applies to your research, read the appropriate section before selecting a response.

Materials & experimental systems

n/a	Involved in the study	
☐	☒	Antibodies
☐	☒	Eukaryotic cell lines
☒	☐	Palaeontology and archaeology
☒	☐	Animals and other organisms
☒	☐	Human research participants
☒	☐	Clinical data
☒	☐	Dual use research of concern

Methods

n/a	Involved in the study	
☒	☐	ChIP-seq
☒	☐	Flow cytometry
☒	☐	MRI-based neuroimaging

Antibodies

Antibodies used	All antibodies are provided with vendor and catalog numbers in the Methods Section.
Validation	CTRP5 antibodies were validated against recombinant CTRP5 and IF localization was confirmed by the use of an additional CTRP5 antibody.

Eukaryotic cell lines

Policy information about cell lines

Cell line source(s)	iPSCs derived from human fibroblasts (2 iPSC clones per donor, 4 human donors from a family with L-ORD)
Authentication	Karyotyping, 3-germ layer differentiation, expression of pluripotency markers, short tandem repeat analysis
Mycoplasma contamination	All cell lines tested negative for mycoplasma contamination on a biweekly basis.
Commonly misidentified lines (See ICLAC register)	*Name any commonly misidentified cell lines used in the study and provide a rationale for their use.*
